# Nostrums

**Published:** 1872-06

**Authors:** 


### NOSTRUMS.

The Code of Medical Ethics says that "every individual, on entering the profession, incurs an obligation to exert his best abilities to maintain its dignity and honor, to exalt its standing, and to extend the bounds of its usefulness." He should contribute the result of his medical observations and experience in a scientific manner, contribute to the usefulness of the profession, and to the well-being of society. There is a moral obligation which binds all to add something, be it little, to the common treasury of knowledge. Having received much from predecessors and contemporaries, is it meritorious, or even excusable, to give nothing in return? Had the past thus "hid its light under a bushel" the present would be groping its way in darkness. Again, it is said that it is "derogatory to professional character for any physician to hold a patent for any surgical instrument or medicine, or to dipense a secret nostrum, whether it be the composition or exclusive property of himself or of others." For, if such nostrums be of real efficacy, any concealment regarding it is inconsistent with benificent and professional liberality; and if mystery alone gives it value and importance, such craft implies either disgraceful ignorance or fraudulent avarice. It is also reprehensible for physicians "to give certificates attesting the value of patent or secret medicines, or in any way to promote the use of them." The Code is the law of the profession, and it asserts that the whole system of manufacturing and vending quack medicines is founded on fraud, and averse alike to science and philanthropy. We believe this is the settled opinion of the profession, and that every one is prepared with facts to make good the assertion. In the first place, there is seldom any correspondence between the composition of patent medicines, as alleged by the proprietors, and the *real* composition. Dr. Solomon, of Liverpool, said of his "Balsam of Gilead," that it had been sanctioned by the most learned physicians of the age; that in the analyzation they had discovered no metal except gold, "pure *virgin gold*, and the true *balsam* of Mecca." Hence, he says this cordial is inestimable, its qualities salubrious, and its preparation the most difficult and costly of all others in chemistry. All this is notoriously false; his balm of Mecca was *brandy*, and his portable *gold*, the flavor communicated by cardamon seeds. The celebrated physicians were of his own kidney; the less distinguished and envious ones, in his eyes, were of course those who would not be duped by his knavery. An apothecary in London sold with no small profit a liquid in bottles which, he alleged, was the *real* Bethesda water. It was taken from a pond in the city, and had some impregnation of sulphur.

To these names we might add our series here at home, from steaming "Thompson" to Panacea "Sevain;" from Catholicon "Potter" to California Vinegar Bitters "Walker;" from "Sanford's" Liver Invigorator to "Simmons'" Liver Regulator; from "Hepsidam's Eureka Life-Blood Elixer" to the "Elixer of Life;" from "Fahnstock's Vermifuge" to the "Worm Destroying Lozenges;" from "Dromgould's Female Bitters" to the "*Bitter of Sad Reflections;*" to say nothing of the shocking absurdity of the *vegetable* pill tribe, which, like a sort of epidemic Diarrhœa, has tormented the intestinal canal of thousands of our far-seeing compatriots, until the American population have become hardened in purgation. All equally well supported by certificates, and having the same zealous regard for the good of their fellow creatures; and all equally indifferent to filthy lucre, themselves testifying. These are a few specimens among hundreds of similar cases of impositions.

In the second place, the practice is frandulent on which, by the plea that patent medicines are compounded of expensive and rare substances, most exorbitant prices are asked for them, when in fact they are often the commonest and cheapest articles.

Third, free inquiry into the properties of a nostrum is prevented, and fraud indirectly avowed, by the proprietor concealing its composition.

Fourth, the publication of cure alone, and sedulously withholding all the cases of failure or injury from the use of such medicines, is deceitful and misleading. What other language can we use, in speaking of the conduct of a man who advertises and lauds, by every possible means, as a sovereign cure for obstinate diseases, an article which he *knows* to be inert? Can we designate by a better title than knave one who mixes with inert substances, for the sake of disguise, a well known medicine, and then ushers it before the public as a discovery of his own and possessing a healing power over diseases, in which the experience of thousands of physicians for centuries, had shown it to be of doubtful if not injurious operation? Is he to be pitied for his ignorance, or reprehended for his daring disregard of the lives of his fellow-men? What pretensions has that imprudent charlatan to be called a public benefactor, and his medicine a public blessing, when he refuses to make its nature or its composition known so that, for one person who now buys it at a high price, hundreds might have the privilege of obtaining it from any apothecary in the country at a trifling expense, if it were really found to be beneficial in disease, as he alleges?

I cannot conceive how a physician could sustain himself as a man of reputation among his professional brethren, or of respectability in general society, who should dare to attempt such a course of concealment as to withhold either the name of the medicine which he alleges to have used with success, or the failures, or occasional bad effects attending its administration. We are members of a liberal profession, among whom there has ever been a most entire community of knowledge. Think of the great and good who, in shedding lustre on their profession, have enobled human nature by a self-denial and disinterestedness for which worldly honors and applause could never be an adequate requital. The secrets of a craft, the tricks of epiricism, cannot weigh with the lofty maxims of true disciples of Hipocrates, who preferred devoting his services to his country—ravaged with a plague—to receiving the glittering treasures and promised honors of the King of Persia.

It is due not only to our feelings of self respect, but also the interests of humanity, that we should stand by the Code of our profession, and put in requisition all the talent and energy we may possess to keep off vantage-ground all impiricism, and lend no countenance to the dissemination of their pernicious heresies.

